# Hard Ticks (Ixodidae) from Wildlife in Liguria, Northwest Italy: Tick Species Diversity and Tick-Host Associations

**DOI:** 10.3390/insects13020199

**Published:** 2022-02-14

**Authors:** Annalisa Accorsi, Irene Schiavetti, Valeria Listorti, Monica Dellepiane, Chiara Masotti, Carlo Ercolini, Lisa Guardone, Elisabetta Razzuoli

**Affiliations:** 1 Struttura Semplice Section of Genoa-Portualità, Istituto Zooprofilattico Sperimentale del Piemonte, Liguria e Valle d’Aosta, 16129 Genoa, Italy; annalisa.accorsi@izsto.it (A.A.); valeria.listorti@izsto.it (V.L.); elisabetta.razzuoli@izsto.it (E.R.); 2 Section of Biostatistics, Department of Health Sciences, University of Genoa, 16100 Genoa, Italy; irene.schiavetti@gmail.com; 3 Unità Operativa Laboratorio di Savona, Struttura Semplice Ponente Ligure, Istituto Zooprofilattico Sperimentale del Piemonte, Liguria e Valle d’Aosta, 17100 Savona, Italy; monica.dellepiane@izsto.it; 4 Struttura Semplice Levante Ligure, Istituto Zooprofilattico Sperimentale del Piemonte, Liguria e Valle d’Aosta, 19136 La Spezia, Italy; chiara.masotti@izsto.it (C.M.); carlo.ercolini@izsto.it (C.E.)

**Keywords:** epidemiology, surveillance, monitoring, vectors

## Abstract

**Simple Summary:**

Wildlife may carry ticks that can transmit pathogens to domestic animals and humans. Tick species from hunted and accidentally found dead wild animals were identified in the framework of a Regional Plan of Monitoring and Surveillance of Wildlife health in the Liguria region, northwest Italy. The results are presented to provide updated information on the tick species in the region and on their host preference. A total of 819 ticks, removed from 259 animals, were found and identified as belonging to five different species. *Ixodes ricinus* was the most frequent species, especially in roe deer and fallow deer, whereas *Dermacentor marginatus* was more specifically associated with wild boar. Wild animals are useful for characterizing and monitoring the tick population in an area, and these data will help in structuring control strategies.

**Abstract:**

Hard ticks’ geographical distribution and abundance are influenced by wildlife population. This work presents the results of the identification of ticks retrieved from wild animals in the framework of a Regional Plan of Monitoring and Surveillance of Wildlife health. The frequency of distribution of ticks in different hosts and their geographical patterns were also investigated. Ticks were collected from game animals (*Sus scrofa*, *Capreolus capreolus*, *Dama dama*, and *Rupicapra rupicapra*) during two hunting seasons (2018–2019 and 2019–2020) in the four provinces of the Liguria region in northwest Italy. In the same period, ticks were also collected from carcasses of *Vulpes vulpes*, *Canis lupus*, *Meles meles*, and *Asio otus* received for necropsy. Tick species were identified according to taxonomic keys. A total of 819 ticks, removed from 259 animals, were found and identified. Overall, *Ixodes ricinus* was the dominant species (62.6%), followed by *Dermacentor marginatus* (24.5%), *Rhipicephalus sanguineus s.l.* (12.5%), *Haemaphysalis punctata* (0.2%), and *Ixodes hexagonus* (0.1%). *I. ricinus* was also the prevalent species in roe deer and in fallow deer and the only species collected from the three wolf carcasses examined. In contrast, *D. marginatus* was the dominant species in *S. scrofa*. This last tick species was also more frequent in one province (Imperia), whereas *Ixodes* spp. were more common in another one (Savona). Wild animals proved to be useful for characterizing and monitoring tick population.

## 1. Introduction

Hard ticks (Ixodidae family) are ectoparasites of domestic and wild animals as well as humans. They are able to transmit viral, bacterial, and parasitic infections, commonly referred to as tick-borne diseases (TBDs) [1]. The geographical distribution and abundance of ticks are influenced by several factors, such as host population dynamics [2], and wild animals may have a great impact on tick epidemiology [3,4]. Additionally, anthropogenic factors, including habitat changes, deforestation, globalization, international animal movements, urbanization, and climate changes may have relevant roles in the emergence and spread of ticks and their pathogens [5,6].

With approximately 40 different species [7], Italy has one of the most diverse tick fauna across Europe [8]. A high species richness and abundance of free-living ticks was recently observed in southern Italy, with up to nine species (*Dermacentor marginatus, Haemaphysalis concinna, Haemaphysalis inermis, Haemaphysalis parva, Haemaphysalis sulcata, Hyalomma marginatum, Ixodes ricinus, Rhipicephalus bursa*, and *Rhipicephalus turanicus*) in a single forested area [9], whereas a lower species richness in the environment was found in central and northern Italy [10,11,12]. *I. ricinus* was generally the dominant species [9,11,12,13,14,15]. Research interest in ticks’ ecology mainly focused on species that have a role in the transmission of pathogens to humans. However, there is a growing body of research on species of ticks that can affect animal health and production and an interest in determining the eco-epidemiological patterns of their transmission cycles, the areas in which ticks develop permanent populations, and how these patterns change through time [2,16]. A baseline of data regarding which tick species circulate in a territory is an essential requirement to provide a basic epidemiological background, to characterize the risk, and to implement control strategies [16,17,18]. Indeed, tick control strategies should be based on information regarding tick spatiotemporal distribution, climate niches, and host preferences [19].

Wild animals may be useful for characterizing and monitoring tick populations, and surveys on wildlife ticks’ abundance and spatial distribution have been conducted in Europe and Italy [3,11,20,21]. However, data often rely on surveys concerning single tick species and their pathogens [15,22] or on ticks collected from the environment [9,10,12,14]. Data regarding hard-tick diversity and tick–host associations are particularly scarce in wild carnivores [3], and only sporadic data are available for some areas of southeastern Europe [9,23].

Wildlife may have a role in the spread of infectious diseases and thus should be subjected to health surveillance [24,25]. In addition to EU regulations [25], in Italy, the National law 157/1992 [26], which defines wildlife as belonging to the state and establishes its protection, also recommends sanitary controls to be implemented. In particular, the regions can issue local regulations on the management and protection of all wildlife species and can activate control plans to ensure the sanitary monitoring of their territory. The surveillance of the health status of the wild fauna in a given territory is a fundamental activity of the authorities responsible for the protection of public and animal health. In addition, hunted game that becomes part of the human food chain must be subject to health surveillance, as established by European Community regulations [27,28,29]. Knowledge on the health status of wildlife is therefore essential, especially because wild animal populations are increasing throughout Europe [30].

Epidemiological surveillance is intended to provide a comprehensive view of the health status of animal populations, as well as to ensure that effective risk assessment can be carried out. According to the Regional Plan of Monitoring and Surveillance of wildlife of the Liguria Region, which is based on guidelines provided by an agreement between the Italian Ministry of Health and the regions and autonomous provinces, a large range of analysis should be performed on different organs and tissues of hunted game, as well as on wild carnivores and birds accidentally found dead. In addition to surveillance of infectious diseases, the Regional plan also includes the search for, and isolation, collection, identification, and analysis of ectoparasites found on these animals, which are conferred to the Istituto Zooprofilattico Sperimentale of Piedmont, Liguria and Aosta Valley (IZSPLV), regional based laboratories of the Ministry of Health, devoted to official controls and research activities. The aim of this work is to present the results of the identification of ticks collected from wild mammals in Liguria over two years of the above noted Regional Plan, and to investigate frequency distribution of ticks in different hosts, as well as the geographical distribution of tick species across the regional territory. Moreover, the influence of human population density and seasonal variations on ticks’ presence were investigated, although this last aspect could be influenced by the different hunting season of the hunted species.

## 2. Materials and Methods

### 2.1. Study Area

Liguria is a region of northwest Italy facing the Ligurian Sea on the south. Its territory (5416 km^2^, counting 1.524.826 inhabitants in 2020; density 282 ab./km^2^) is mainly occupied by mountains and hills, with very limited plain areas. The western part of the region (“Riviera di Ponente”, west of Genoa) is occupied by the Maritime Alps, with Mount Saccarello, the highest peak, reaching 2200 m. In the central and eastern section of the region are the Ligurian Apennines, with Mount Maggiorasca (1799 m) near the border with the Emilia-Romagna region. Colle di Cadibona (435 m above sea level) is the connection point between the Maritime Alps on the west, and the Ligurian Apennines on the east. The limited inland extension of the regional territory, which at no point exceeds a distance of 35 km from the sea, does not allow the presence of proper rivers flowing into the Ligurian Sea, but only of short seasonal streams [31,32]. The Ligurian Sea, already very deep even at a short distance from the coast, exerts an extraordinary mitigating action on the climate; additionally, the mountains form a barrier against cold winds coming from the north. The western province, Imperia, has the mildest climate (average temperatures: 9 °C in January, 25 °C in July), whereas the average winter temperature is slightly lower (7 °C) in Savona and Genoa provinces, which are less protected by the lower Apennines altitude. Rainfall is more abundant in Genoa and in the eastern part of the region (1000–1500 mm/year). The climatic conditions vary from the hills to the higher mountainous areas, although a further distinction must be made between the milder side, facing the sea, and the side facing the Po Valley, where significantly lower average temperature (0 °C in January) and maximum rainfall (>2000 mm/year) are observed [31]. Four typical plant associations can be identified: at the lower altitude the Mediterranean scrub, rich in holm oaks, laurels, oleanders, myrtles, rosemary, heather, and olive trees. Moving away from the coast, pine and chestnut woods are found up to 800 m of altitude, followed by beech, up to 1500 m, and finally larch and fir woods. The forest covers over 50% of the total area of the region, sustaining populations of wild boar, roe deer, and red deer, the most common wild animals in Liguria [33].

### 2.2. Ticks Collection and Identification

**Figure 1:**
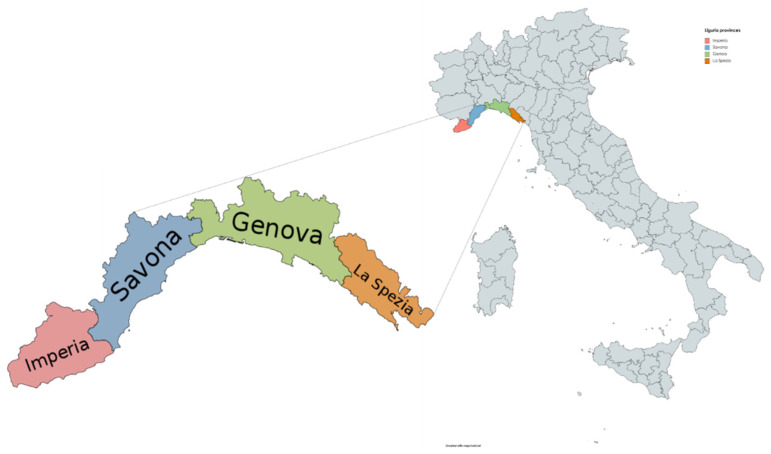
Map of Liguria region with the four investigated provinces (study area).

Ticks were collected from game animals during two consecutive hunting seasons (2018–2019 and 2019–2020) as required by the Regional Plan of Monitoring and Surveillance of wildlife in Liguria Region, northwest Italy. Targeted game species were wild boar (*Sus scrofa*), roe deer (*Capreolus capreolus*), fallow deer (*Dama dama*), and chamois (*Rupicapra rupicapra*). It should be noted that the hunting period (defined by regional laws) may vary for each species. In detail: hunting sessions occurs from October to January for wild boar, September–December for chamois, January–March for fallow deer, and January–April and June for roe deer. Ears, or more rarely, portions of skin of hunted game were delivered to the IZSPLV from each of the four provinces of the Liguria Region, i.e., Imperia (IM), Savona (SV), Genova (GE), and La Spezia (SP) (Figure 1), thanks to the collaboration with local hunting associations. Each anatomical portion was univocally identified and thoroughly inspected to detect the presence of ticks (wild boars: anatomical portions from 1828 examined individuals; roe deer: n = 276; fallow deer: n = 36; chamois: n = 4). Furthermore, in the same timeframe, ticks were also collected from carcasses of wild animals hunted, road-killed, or found dead and received for necropsy, including fox (*Vulpes vulpes*), wolf (*Canis lupus*), European badger (*Meles meles*), and long-eared owl (*Asio otus*). If needed, anatomical samples and carcasses were stored at −20 °C before the analysis. Ticks were carefully removed with forceps and morphologically examined under a stereoscopic microscope to achieve species level identification according to taxonomic keys [34,35].

### 2.3. Database and Statistical Analysis

Data regarding date of collection, host species, geographical origin (town hall and province level), species, and sex of the identified ticks were organized in an Excel database and used for statistical analysis.

All data were presented in a descriptive manner as median with range (minimum–maximum) for continuous variables, and number with percentage for categorical variables, as appropriate. Missing data were not replaced. Differences in distributions of ticks (overall or within each host) between two hunting seasons were investigated with Chi square test or Fisher’s exact test, as appropriate.

## 3. Results

At least one tick was found on each of a total of 259 wild animals. Details of the number of wild host individuals found positive per hunting season are given in Table 1. Overall, 819 tick specimens were found, belonging to five different species. *Ixodes ricinus* (n = 513, 62.6% of the overall collected ticks) was the dominant species, followed by *Dermacentor marginatus* (n = 201, 24.5%) and *Rhipicephalus sanguineus s.l.* (n = 102, 12.5%), whereas *Haemaphysalis punctata* (n = 2, 0.2%) and *Ixodes hexagonus* (n = 1, 0.1%) were only found occasionally. Details are given in Table 2. The prevalence rate (presence of at least one tick) was calculated only for the three main host species sampled during hunting sessions (wild boar, roe deer, and fallow deer). The overall prevalence in wild boar was 4.3% (78 positive samples out of 1828 examined), whereas for roe deer it was 50.0% (138 positive samples out of 276 examined) and for fallow deer 47.2% (17 positive samples out of 36 examined). The prevalence was not calculated on the other species, due to their low numbers (*R. rupicapra*) or, in the case of hosts accidentally found dead and sampled at necropsy (*V. vulpes*, *C. lupus lupus, M. meles*, and *A. otus*), due to the fact that their frequent bad conservation status prevented the accurate collection of ticks, which might also have left the host, making a negative result not fully reliable.

The distribution of tick species in the different hosts is presented in Table 1. The dominant tick species, *I. ricinus*, was found in all the examined host species with the only exception being the single badger carcass. However, its frequency varied in the different species. It was the dominant species in roe deer and fallow deer, where it represented 93.1% and 63.0% of the ticks collected on those hosts, respectively. It was also the only species identified out of the 33 tick specimens found on the three wolf carcasses examined, and the only tick identified on the owl. In contrast, *I. ricinus* was less frequent on chamois, foxes, and wild boars. Regarding this last host, the dominant tick species was by far *D. marginatus*; 100% of the 201 ticks identified as *D. marginatus* collected were found on wild boars, where it accounted for 96.2% of the total collected ticks. The other tick species found on wild boars were *I. ricinus* (1.9%), *R. sanguineus s.l.* (1.4%), and the only specimens of *I. hexagonus* found in the study. *H. punctata* was identified only on fallow deer, in both hunting seasons (Table 1). As shown in Table 1, the probability of finding *R. sanguineus s.l.* in roe deer in season 2019–2020 was 16 times higher than in the previous season.

**Figure 2:**
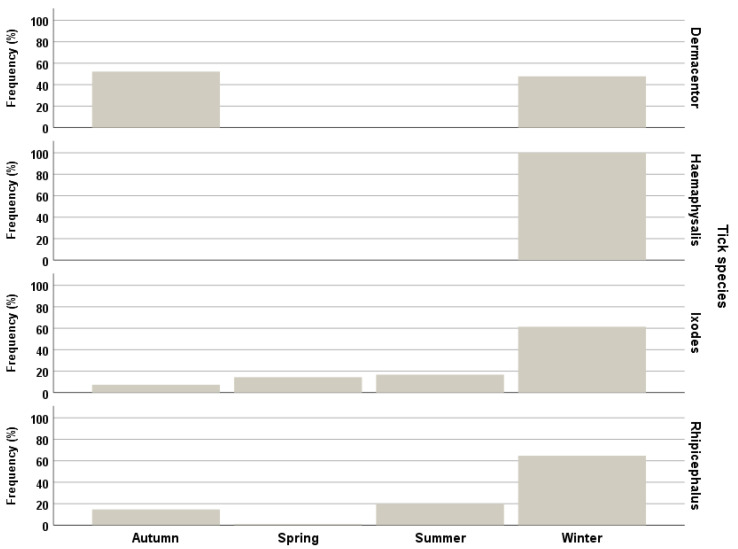
Seasonal pattern of the overall frequency of the collected tick genus.

**Figure 3:**
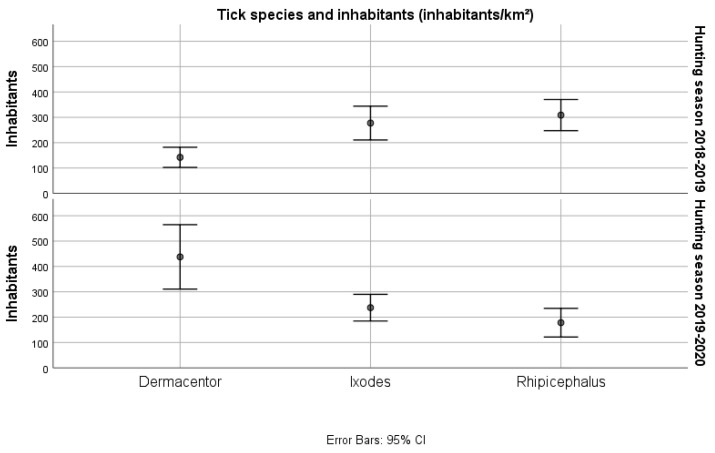
Relationship between the presence of tick genus and inhabitants’ density in the area of host collection (townhall level).

The median number and range of ticks by host species is presented in Table 3. The highest number of total ticks were observed in fallow deer, followed by wolf and fox, chamois, and roe deer. Furthermore, the distribution of ticks in wild hosts in the four provinces is presented (Table 4). The frequency of *D. marginatus* was found to be higher in Imperia province, as 75.6% of the ticks of this species were found there, whereas *Ixodes* spp. were more frequent in Savona province. In fact, 80% of the ticks of this genus were found in this latter province, where also the frequency of *R. sanguineus s.l.* was higher (61.8%) compared to the other provinces. These observations could partly be explained by differences in the sampled hosts in the four provinces: most of the examined wild boars were sampled in Imperia (Table 4). Seasonal patterns of the overall tick frequency (Figure 2) and the relationship between the presence of a tick genus and inhabitants’ density (Figure 3) were also investigated.

## 4. Discussion

Liguria region is cover by mountains for over 65% of its territory and by hills for most of the remaining surface [32], favouring the presence of wild animal species, mainly wild boars and wild ruminants [33], but an increasing presence of wolves is also reported [36]. The regional geological conformation and the short distance between the coastal and mountainous areas favour contacts between humans, domestic animals, and wild animals, which more and more frequently are found near the villages and urban centres. In recent decades, the increasing proximity to humans has transformed ungulates, in particular wild boar, into almost synanthropic animals. Thus, the likelihood of exposure to wildlife ticks may increase, as suggested for other countries [16]. A few previous studies on ixodid ticks conducted in Liguria are available. In 1995 and 1996, 318 ticks were recovered from 240 people in one of the four provinces (Savona). Most of the ticks were *I. ricinus* (89.3%), followed by *R. sanguineus* (9.8%) and *D. marginatus* (0.9%) [37]. A subsequent study collected 1,464 questing ticks during monthly dragging sessions from March to August 2011 in three provinces (Imperia, Genoa, and La Spezia) of the Liguria region, northwestern Italy, evaluated the species occurrence, spatial distribution, and relative abundance, confirming *I. ricinus* as the dominant species [38]. More recently, a study investigated pathogens in 200 ticks (124 *Ixodes*, 68 *Dermacentor*, 34 *Haemaphysalis*, and 9 *Rhipicephalus* spp.) collected from 49 roe deer, 41 wild boars, 7 chamois, 2 fallow deer, and 1 marten (*Martes foina*), and found 36 ticks positive for *Rickettsia* spp. (32 *I. ricinus* and 4 *D. marginatus*) and 3 for *Anaplasma* spp. (2 *H. punctata* and 1 *I. ricinus*) by PCR, whereas all samples were negative for *Borrelia* spp. and tick-borne encephalitis virus (TBEv) [39]. Thus, the current results on the identification of tick species collected from hunted game or on wildlife accidentally found dead, obtained in the framework of the Regional Plan of Monitoring and Surveillance of wildlife in Liguria Region, can help update the existing information and establish tick–host associations in the study area.

*Ixodes ricinus* was the most abundant species in this survey, in agreement with another study on questing ticks in the region [38] and with several other Italian and European studies [9,11,12,13,14,15,17]. This species, also known as “wood”, “sheep”, or “castor-bean” tick, is also the most frequently reported Ixodidae to bite humans in Europe [40], and it is considered the main TBDs vector in Europe [4]. It can transmit viral, bacterial, and protozoan agents, such as the TBEv, *Borrelia burgdorferi* sensu lato (s.l.), *Rickettsia*, *Anaplasma*, and *Babesia* spp., to domestics and wild animals, as well as humans [15,41]. The suitability of this species as a vector of several pathogens is favoured by its large host spectrum, being able to feed on more than 300 animal species [40]. As noted, in our study this species was mainly collected from roe deer and fallow deer (Table 1). The occurrence of *I. ricinus* on roe deer in Liguria is in agreement with historical data [37], when a highest frequency of *I. ricinus* human bites in May, June and July and in the municipalities of Savona province, where roe deer density was highest, was also observed. Nevertheless, in our study *I. ricinus* was also found on five other species (wild boar, chamois, wolf, fox, and owl), in agreement with the observations of Maioli et al. [12] who found *I. ricinus* on several vertebrate species (roe deer, wild boar, red deer, and European brown hare) in the Emilia Romagna region.

Noteworthy is our finding of *I. ricinus* in wolves, where it was the only tick species found, different from foxes. This tick species was already found on wolves in Romania [3 and references therein], whereas *D. marginatus* and *Ixodes acuminatus* were found in southern Italy [21]. However, data on ticks and related pathogens on wolfs are scarce, as, among wildlife, the wild carnivores are particularly difficult to monitor and capture. However, due to their wide home range, they are exposed to many tick species and of particular interest for parasite ecology [3,42].

*Ixodes ricinus* is currently expanding its geographical range [4], and it has been increasingly reported also in urban green areas, where the likelihood of exposure to tick bites may be high for human beings and companion animals [14,41]. In Italy, it is known to occur throughout the peninsula, with the highest density in hilly and pre-alpine northern areas, characterized by a temperate climate, with cold winters and cool and humid summers [15]. However, *I. ricinus* was also found as the most abundant species in south Italy, showing that the southern Italian climate, with hot and dry summers, is suitable for its development [13]. Indeed, also in the present study, it was the only tick species found in all four provinces (Table 4) and throughout the year (Figure 2). However, it must be noted that the results concerning the year distribution might be affected by the hunting sessions, which differ for the different game species. This aspect might also explain the higher overall tick prevalence observed in roe deer and fallow deer (50.0% and 47.2%, respectively), which are also hunted in spring (see Section 2.2), compared to wild boar, which is only hunted from October to January.

Regarding the other *Ixodes* species found, *I. hexagonus,* also known as the hedgehog tick, appeared to be much rarer in the investigated hosts, as is was found only once on a wild boar. The occurrence of this species in Liguria was already observed in a previous study conducted in the region [37]. This species is reported throughout Europe due to its nidicolous behaviour [43], allowing its establishment in an area independently from weather conditions [16].

*Dermacentor marginatus*, the ornate sheep tick, is known to occur throughout southern Europe [44]. In this study it was only found on wild boar, thus suggesting a tick–host association, as already observed in several studies [12,22,45]. For instance, *D. marginatus* was found in 99.4% of 93 wild boars examined from the Campania region, southern Italy, whereas *I. ricinus* was only identified in 0.6% of them, suggesting that wild boars are involved in the maintenance of *D. marginatus* in the environment and advocating an integrated management approach for wild boar population control and TB diseases prevention in animals and humans [22]. *Dermacentor* spp. are open-country tick species that are considered emerging vectors in Europe; whereas *D. marginatus* is rather common in northern Italy, as confirmed by our results, and *D. reticulatus* is typical of central Europe, where it is reported in urban and suburban areas [44]. Its occasional collection south of the Alps, including Turin hillside, was recently reported [45], but according to our results this species does not seem to be present in Liguria.

A great plasticity and adaptability to different environments has been reported for *D. marginatus,* which was found in different habitats, up to 1600 m asl [46]. Although this species is less frequently reported to bite humans compared to more generalist species such as *I. ricinus*, two *D. marginatus* collected on human patients at a local emergency unit in Piedmont were recently analysed. The finding may suggest a significant presence of the species in the area, and it is particularly relevant considering that both tick specimens were found to be positive for *Rickettsia slovaca* [45].

In our study *R. sanguineus s.l.,* the so called “brown dog tick” due to its common occurrence on dogs [18], was mainly found on fallow deer, followed by roe deer, but it was also collected from chamois, foxes, wild boar, and badger, showing a wide host range. Regarding carnivores, it is worth noting that *R. sanguineus* was collected from foxes but not from wolf carcasses. In Europe, several ticks of the genera *Ixodes*, *Dermacentor*, *Haemaphysalis*, *Hyalomma*, and *Rhipicephalus* were collected from foxes. In Germany, for instance, *I. ricinus*, *I. hexagonus*, *I. canisuga*, and *I. kaiseri* were commonly collected from foxes, whereas *D. reticulatus* and *H. concinna* were only occasionally found [47]. A study in southern Italy found the following species on 6 *V. vulpes* out of 81 examined: *D. marginatus*, *Haemaphysalis erinacei*, *Ixodes canisuga*, *I. ricinus*, *Rhipicephalus bursa*, and *R. turanicus* [21].

A recent spread and changes in the distribution pattern were also reported for *R. sanguineus s.l.* [16]. This species appears to be moving from the Mediterranean basin to more northern latitudes [23].

Only two specimens of *H. punctata* were found in this study, both on fallow deer. In contrast, *H. punctata* was dominant in Monti Sibillini National Park (central Italy), where almost all (98.9%) the specimens collected by dragging belonged to this species, whereas only 0.5% and 0.6% were *I. ricinus* and *D. marginatus*, respectively [10].

## 5. Conclusions

Ticks are arthropods of great medical and veterinary interest. Although TBDs are considered rare in Europe, the geographical expansion of ticks and the increasing number of reported tick bites highlight the relevance of such issues [14]. The current results on the identification of tick species from hunted game or on wildlife accidentally found dead contribute to updating existing information and establishing tick–host associations in the study area. The results confirm the dominance of *I. ricinus* and its wide host range, as well as a widespread diffusion of *D. marginatus,* particularly associated with wild boars. The occurrence of pathogens in these tick species has already been reported in the same [39] and in neighbouring regions, such as Piedmont [41,45]. This scenario poses new challenges in terms of diagnosis, treatment, and control, highlighting the need for a One Health approach towards a better management of these infections in animals and humans [19]. In this light, systematic surveillance remains essential to provide a complete overview of ticks in a given area. The raising number of free-living wild ungulates, especially red deer, roe deer, and wild boar, in the study area and across Europe, and anthropogenic changes of the environment favouring the diminishing of the boundaries between wild and domestic animals, may increase the exposure of both animals and humans to infective agents, including tick-borne pathogens.

## Figures and Tables

**Table 1:** Frequency of the collected tick species in the wild hosts during two hunting seasons. HS1: Hunting Season 1: 2018–2019; HS2: Hunting Season 2: 2019–2020.

Hosts	N Hosts Found Positive for the Presence of at Least One Tick (% over the Total Host)				N of Collected Ticks (% over the Total Collected Ticks in Each Host)			*p*
	HS1	HS2	Tot	Tick Species	HS1	HS2	Tot	
Roe deer	88 (52.4)	50 (54.9)	138 (53.3)	*Ixodes ricinus*	275 (98.6)	100 (80.6)	375 (93.1)	<0.001
				*Rhipicephalus sanguineus s.l.*	4 (1.4)	24 (19.4)	28 (6.9)	
				Total ticks	279	124	403	
Wild boar	60 (35.7)	18 (19.8)	78 (30.1)	*Dermacentor marginatus*	154 (95.7)	47 (97.9)	201 (96.2)	0.93
				*Ixodes ricinus*	3 (1.9)	1 (2.1)	4 (1.9)	
				*Rhipicephalus sanguineus s.l.*	3 (1.9)	0 (0.0)	3 (1.4)	
				*Ixodes hexagonus*	1 (0.6)	0 (0.0)	1 (0.5)	
				Total ticks	161	48	209	
Fallow deer	17 (10.1)	16 (17.6)	33 (12.7)	*Ixodes ricinus*	56 (56.6)	41 (74.5)	97 (63.0)	0.06
				*Rhipicephalus sanguineus s.l.*	42 (42.4)	13 (23.6)	55 (35.7)	
				*Haemaphysalis punctata*	1 (1.0)	1 (1.8)	2 (1.3)	
				Total ticks	99	55	154	
Chamois	2 (1.2)	1 (1.1)	3 (1.2)	*Ixodes ricinus*	2 (40.0)	0 (0.0)	2 (25.0)	0.46
				*Rhipicephalus sanguineus s.l.*	3 (60.0)	3 (100.0)	6 (75.0)	
				Total ticks	5	3	8	
Wolf	0 (0.0)	3 (3.3)	3 (1.2)	*Ixodes ricinus*	0 (0.0)	33 (100.0)	33	/
Fox	0 (0.0)	2 (2.2)	2 (0.8)	*Ixodes ricinus*	0 (0.0)	1 (12.5)	1 (12.5)	0.99
				*Rhipicephalus sanguineus s.l.*	0 (0.0)	7 (87.5)	7 (87.5)	
				Total ticks	0	8	8	
Badger	0 (0.0)	1 (1.1)	1 (0.4)	*Rhipicephalus sanguineus s.l.*	0 (0.0)	3 (100.0)	3	/
Owl	1 (0.6)	1 (0.0)	1 (0.4)	*Ixodes ricinus*	1 (100.0)	0 (0.0)	1	/
Overall			259		545	274	819	

Results are expressed as count and percentage—n (%).

**Table 2:** Tick species found in the two hunting seasons.

		Hunting Season 2018–2019	Hunting Season 2019–2020	Overall
*Ixodes ricinus*	Females	330 (97.9)	157 (89.2)	487
	Males	7 (2.1)	19 (10.8)	26
	Total	337	176	513 (62.6)
*Dermacentor marginatus*	Females	77 (50.0)	28 (59.6)	105
	Males	77 (50.0)	19 (40.4)	96
	Total	154	47	201 (24.5)
*Rhipicephalus sanguineus s.l.*	Females	50 (96.2)	50 (100.0)	100
	Males	2 (3.8)	0 (0.0)	2
	Total	52	50	102 (12.5)
*Haemaphysalis punctata*	Females	1 (100.0)	0 (0.0)	1
	Males	0 (0.0)	1 (100.0)	1
	Total	1	1	2 (0.2)
*Ixodes hexagonus*	Females	1 (100.0)	0 (0.0)	1
	Total	1	0	1 (0.1)
Overall				819

Results are expressed as count and percentage—n (%).

**Table 3:** Median number and range of tick genera per host species.

		Hunting Season 2018–2019	Hunting Season 2019–2020
Total hosts	*Rhipicephalus*	0 (0–14)	0 (0–20)
	*Ixodes*	1 (0–17)	1 (0–25)
	*Dermacentor*	0 (0–24)	0 (0–15)
	*Haemaphysalis*	0 (0–1)	0 (0–1)
	Total ticks	2 (1–24)	2 (1–25)
Roe deer	*Rhipicephalus*	0 (0–3)	0 (0–20)
	*Ixodes*	2 (0–12)	2 (0–5)
	*Dermacentor*	0 (0–0)	0 (0–0)
	*Haemaphysalis*	0 (0–0)	0 (0–0)
	Total ticks	2 (1–12)	2 (1–20)
Wild boar	*Rhipicephalus*	0 (0–2)	0 (0–0)
	*Ixodes*	0 (0–1)	0 (0–1)
	*Dermacentor*	1 (0–24)	1 (0–15)
	*Haemaphysalis*	0 (0–0)	0 (0–0)
	Total ticks	1 (1–24)	1 (1–15)
Fallow deer	*Rhipicephalus*	0 (0–14)	0 (0–12)
	*Ixodes*	2 (0–17)	3 (0–6)
	*Dermacentor*	0 (0–0)	0 (0–0)
	*Haemaphysalis*	0 (0–1)	0 (0–1)
	Total ticks	5 (1–17)	3 (1–12)
Chamois	*Rhipicephalus*	2 (0–3)	3 (3–3)
	*Ixodes*	1 (0–2)	0 (0–0)
	*Dermacentor*	0 (0–0)	0 (0–0)
	*Haemaphysalis*	0 (0–0)	0 (0–0)
	Total ticks	3 (2–3)	3 (3–3)
Wolf	*Rhipicephalus*		0 (0–0)
	*Ixodes*		4 (4–25)
	*Dermacentor*		0 (0–0)
	*Haemaphysalis*		0 (0–0)
	Total ticks		4 (4–25)
Fox	*Rhipicephalus*		4 (0–7)
	*Ixodes*		1 (0–1)
	*Dermacentor*		0 (0–0)
	*Haemaphysalis*		0 (0–0)
	Total ticks		4 (1–7)
Badger	*Rhipicephalus*		3 (3–3)
	*Ixodes*		0 (0–0)
	*Dermacentor*		0 (0–0)
	*Haemaphysalis*		0 (0–0)
	Total ticks		3 (3–3)
Owl	*Rhipicephalus*	0 (0–0)	
	*Ixodes*	1 (1–1)	
	*Dermacentor*	0 (0–0)	
	*Haemaphysalis*	0 (0–0)	
	Total ticks	1 (1–1)	

Results are expressed as median with range (min–max) per host. Missing data mean no collection.

**Table 4:** Host and tick genera in the four different provinces. N refers to host number.

		Genova	Imperia	La Spezia	Savona
Roe deer	(N = 138)	9	18	0	111
	*Ixodes*	25 (100.0%)	60 (75.0%)	0 (0.0%)	290 (97.3%)
	*Rhipicephalus*	0 (0.0%)	20 (25.0%)	0 (0.0%)	8 (2.7%)
Wild boar	(N = 78)	3	62	1	12
	*Dermacentor*	1 (33.3%)	152 (97.4%)	0 (0.0%)	48 (98.0%)
	*Ixodes*	2 (66.7%)	1 (0.6%)	1 (100.0%)	1 (2.0%)
	*Rhipicephalus*	0 (0.0%)	3 (1.9%)	0 (0.0%)	0 (0.0%)
Fallow deer	(N = 33)	1	0	0	32
	*Haemaphysalis*	0 (0.0%)	0 (0.0%)	0 (0.0%)	2 (1.3%)
	*Ixodes*	5 (100.0%)	0 (0.0%)	0 (0.0%)	92 (61.7%)
	*Rhipicephalus*	0 (0.0%)	0 (0.0%)	0 (0.0%)	55 (36.9%)
Chamois	(N = 3)	0	3	0	0
	*Ixodes*	0 (0.0%)	2 (25.0%)	0 (0.0%)	0 (0.0%)
	*Rhipicephalus*	0 (0.0%)	6 (75.0%)	0 (0.0%)	0 (0.0%)
Wolf	(N = 3)	1	0	0	2
	*Ixodes*	4 (100.0%)	0 (0.0%)	0 (0.0%)	29 (100.0%)
Fox	(N = 2)	0	2	0	0
	*Ixodes*	0 (0.0%)	1 (12.5%)	0 (0.0%)	0 (0.0%)
	*Rhipicephalus*	0 (0.0%)	7 (87.5%)	0 (0.0%)	0 (0.0%)
Badger	(N = 1)	0	1	0	0
	*Rhipicephalus*	0 (0.0%)	3 (100.0%)	0 (0.0%)	0 (0.0%)
Owl	(N = 1)	0	1	0	0
	*Ixodes*	0 (0.0%)	1 (100.0%)	0 (0.0%)	0 (0.0%)
Overall	*Ixodes*	36 (97.3%)	65 (25.4%)	1 (100.0%)	412 (78.5%)
	*Dermacentor*	1 (2.7%)	152 (59.4%)	0 (0.0%)	48 (9.1%)
	*Haemaphysalis*	0 (0.0%)	0 (0.0%)	0 (0.0%)	2 (0.4%)
	*Rhipicephalus*	0 (0.0%)	39 (15.2%)	0 (0.0%)	63 (12.0%)

## Data Availability

Not applicable.
